# A Comparative Study of Wound Infections Following Wound Closure Using Staples and Absorbable Sutures Among Obese Patients Undergoing Lumbar Fusion Surgery

**DOI:** 10.7759/cureus.63254

**Published:** 2024-06-26

**Authors:** Akshay P, Vinod Kumar K, Manoj K Ramachandraiah, Arun H Shanthappa

**Affiliations:** 1 Department of Orthopedics, Sri Devaraj Urs Medical College (SDUMC), Kolar, IND; 2 Department of Orthopedics and Trauma, Sri Devaraj Urs Medical College (SDUMC), Kolar, IND; 3 Department of Orthopedics, Sri Devaraj Urs Academy of Higher Education and Research, Kolar, IND

**Keywords:** staphylococcus, infection, suture, staples, wound

## Abstract

Background

Infections of the wounds, organs, or spaces that develop following surgery are known as surgical site infections (SSIs). The incidence of wound infections in obese patients undergoing lumbar spine surgery with the use of absorbable sutures versus staples for skin closure has not been studied previously.

Materials and methods

We conducted a retrospective observational study in our hospital where cases of lumbar spine surgery meeting the inclusion criteria were chosen retrospectively from March 2021 to March 2023. A total of 40 patients aged >18 years and <75 years who underwent lumbar spine surgery were covered by this investigation. Two cohorts with 20 patients in each were chosen from the population. Group A used a skin stapler to close wounds, whereas group B used absorbable sutures. The number of wound infections was the main result. Using SPSS version 23.0 (IBM SPSS Statistics, Armonk, NY), all data were analyzed after being entered into an Excel sheet (Microsoft Corp., Redmond, WA).

Results

A total of 40 participants were included in this study, and it revealed that there was no discernible variation in the groups' mean age or gender distribution. There is a significantly higher incidence of SSI in the absorbable suture group (35%) compared to the staple group (15%). The mean duration in days for the development of SSI in the absorbable suture group (9.86±2.12) was early compared to the staple group (12.67±2.08), which was statistically significant (p<0.05).

Conclusion

Compared to absorbable sutures, the current study showed a decreased incidence of surgical site infection in obese individuals receiving skin staples for wound closure.

## Introduction

Infections of the wounds, organs, or spaces following surgery are referred to as surgical site infections (SSIs) [1]. A catastrophic consequence following spine surgery is surgical site infection (SSI). SSI raises morbidity and mortality and is associated with prolonged hospital stays, reoperations, and long-term IV antibiotic use [2]. In clean orthopedic surgery, the overall incidence of postoperative infections is reported to be between 0.6% and 1.8%, which is comparatively lower than in other areas. However, with lengthy spinal surgery, especially spinal fusion, it is higher; in high-risk individuals, it is said to rise to 4.15% [3]. After spine surgery, the incidence of SSIs varies from 0.2% to 18.8% [4].

Obesity, including morbid obesity, both during and after surgery, is an independent risk factor for problems after spine surgery [5]. Longer operating durations, increased blood loss, greater treatment costs, a higher risk of death, and a higher incidence of surgical site infections have all been associated with obese patients undergoing spine surgery [6].

SSI delays wound healing, thus increasing the duration of hospital stays and the healthcare costs of patients [7-9]. Hence, appropriate infection prevention and control measures to prevent and reduce SSI for different surgical procedures need to be researched. A watertight closure at the skin layer is necessary for wound healing, reducing the risk of surgical site infections, encouraging patient self-care, and determining whether follow-up is necessary [10]. Sutures are typically used to heal subcutaneous sores during spinal fusion. Nevertheless, a variety of alternative techniques, such as suture-free techniques such as topical skin adhesives or skin staples, can be utilized to seal wounds at the skin's surface [11]. The primary objectives of wound closure are to promote early mobilization, decrease the risk of infection, and enable quick skin healing while maintaining a satisfactory cosmetic outcome.

Although there are a number of techniques for closing the skin after spine surgery, it is still unknown which technique is best [12]. Numerous studies have advised against using metal staples to close the skin during orthopedic surgery [13,14]. However, since skin closure procedures are linked to a high rate of postoperative infection, research on their use in obese patients undergoing spine surgery has not yet been done.

We conducted this study to assess, for the first time, wound infections in obese patients following single-level lumbar decompression with instrumented fusion surgery employing absorbable sutures (Monocryl 3-0) and staples for skin closure.

## Materials and methods

Our study was a retrospective, hospital-based observational study. Cases of single-level lumbar decompression and instrumented fusion surgery meeting the inclusion criteria who presented at R.L. Jalappa Hospital and Research Centre were chosen retrospectively from March 2021 to March 2023. Ethical clearance was obtained from the Institutional Ethics Committee of Sri Devaraj Urs Medical College (approval number SDUMC/KLR/IEC/484/2023-24) to conduct the study. The participants in this study were all those who underwent single-level lumbar decompression and instrumented fusion operations between the ages of 18 and 75. Patients with vertebral or spinal tumors, spinal infections such as pyogenic infections or tuberculosis, uncontrolled diabetes, and revisions of spine surgery were excluded from the study.

Analysis was conducted on 40 obese and morbidly obese patients who had single-level lumbar decompression and instrumented fusion. Data were retrieved from patient medical records with a minimum three-month follow-up period. Additional data extracted from electronic medical records (EMRs) included body mass index (BMI), red blood cell count, serum albumin, history of smoking, use of steroids, transfusion, diagnosis, region, surgical approach, instrumentation, estimated blood loss, duration of the procedure, and presence of hypertension, diabetes mellitus, malignant tumor, rheumatoid arthritis, obstructive lung disease, or coronary artery disease. The case sheets were separated into two groups: group A (wound closure with a skin stapler) and group B (wound closure with absorbable sutures (Monocryl 3-0)).

Twenty patients from each group were selected by a computer-based random number generator. Patients were assessed based on data retrieved from picture archiving and communication system (PACS). All patients were operated on by the same surgeon. Two drain tubes, one in the subfascial layer and another in the subcutaneous layer, connected to a single collection unit were used as a standard practice in all patients. The number of wound infections was the main result. An infection of the wound was indicated by erythema, induration, pain, and a culture-positive discharge of serous or contaminated fluid. SSI was often taken into consideration until 90 days after surgery. A superficial SSI was usually identified with complaints of pain, local tenderness, redness, and a local rise in temperature at the surgical site involving skin and subcutaneous tissue. Deep SSI affected the fascia and muscles, presenting with fever and abscess formation along with pain and tenderness. The diagnosis of SSI in patients included in the study was confirmed by drain tip culture growth and ultrasound. Additionally, the incidence of dehiscence was examined. Risk factors for inadequate wound healing or infection include patient age, sex, body mass index, performance level as defined by the American Society of Anesthesiologists, and wound condition as measured by the Centers for Disease Control and Prevention-National Nosocomial Infection Surveillance SSI Risk Index [15].

Statistical analysis

For analysis, SPSS version 23.0 (IBM SPSS Statistics, Armonk, NY), operating on Windows 10, was utilized after all data were entered into an Excel spreadsheet (Microsoft Corp., Redmond, WA). Data were provided as mean, standard deviation (SD), frequency, and percentage and were represented through tables. For the purpose of comparing the mean difference between continuous and categorical data, the unpaired t-test and the chi-square test were utilized. For all intents and purposes of statistics, a p-value of less than 0.05 was considered meaningful.

## Results

Forty obese and severely obese individuals met the inclusion criteria and were included in the study. The patients were split into two groups according to the closure materials, which included staples in one group and absorbent sutures in another. The groups' mean age and gender distribution did not differ significantly from one another (enrolled) (p>0.05).

Out of the total, in group A, 14 and six patients were obese and morbidly obese, respectively. Out of the total, in group B, 16 and four patients were obese and morbidly obese, respectively (Table 1).

**Table 1:** Distribution among study participants based on BMI (n=84) BMI: body mass index

BMI	Obese	Morbidly obese
Group A	14	6
Group B	16	4

The groups' mean age and gender distribution did not differ significantly from one another (enrolled) (p>0.05). The study noted that both staples (65%) and sutures (55%) were commonly done among males, and this difference was not significant (p=0.695) (Table 2).

**Table 2:** Distribution of genders among the groups

		Staples		Sutures		p-value
		Number	%	Number	%	
Gender	Female	7	35%	9	45%	0.695
	Male	13	65%	11	55%	

Among the staple group, the majority of surgical fusion was noted at L3-L4, followed by L4-L5, L5-S1, and L2-L3. In comparison, among the suture group, the majority of fusion was noted at L4-L5, followed by L3-L4 and L2-L3. However, this difference was not statistically significant (Table 3).

**Table 3:** Distribution between the groups L: lumbar vertebra, S: sacral vertebra

Surgical fusion level	Group			
	Staples		Sutures	
	Number	%	Number	%
L2-L3	1	5%	3	15%
L3-L4	8	40%	4	20%
L4-L5	8	25%	13	35%
L5-S1	3	15%	0	0%

Blood parameters did not significantly differ across the groups. The mean time for SSI development in the patients varies significantly between groups (p<0.05). The mean duration of development of SSI in the absorbable suture group (9.86±2.12) was early compared to the staple group (12.67±2.08) (p<0.05) (Table 4).

**Table 4:** Comparison of demographic details of patients between the groups Hb: hemoglobin, WBC: white blood cells, FBS: fasting blood sugar, SSI: surgical site infection, SD: standard deviation

	Staples		Sutures		p-value
	Mean	SD	Mean	SD	
Age	51	0.7	51	0.6	0.291
Hb	12.9	1.8	14.0	2.0	0.71
WBC	7,001	2,644	7,093	2,293	0.90
FBS	103	14	101	16	0.668
Mean duration of SSI development in days	12.67	2.08	9.86	2.12	0.05

There was a significant difference in the presence of SSI between the groups. There was a significantly higher incidence of SSI in the absorbable suture group (35%) compared to the staple group (15%). Also, there was a higher incidence of *Staphylococcus* infection in the suture group compared to the staple group (Table 5).

**Table 5:** Comparison of SSI, infection site, and culture report between the groups SSI: surgical site infection

		Staples		Sutures		Chi-square (p-value)
		Number	%	Number	%	
Type of surgery	Elective	17	85%	18	90%	0.633
	Emergency	3	15%	2	10%	
Presence of SSI	No	17	85%	13	65%	0.04
	Yes	3	15%	7	35%	
Bacteria culture	NA	17	85%	13	65%	0.01
	Negative	1	5%	0	0%	
	Pseudomonas	1	5%	2	10%	
	Staphylococcus	1	5%	5	25%	
Infection site	Deep	1	33.3%	2	28.6%	0.687
	Superficial	2	66.7%	5	71.4%	

Our study also found a statistically significant increase in the incidence of both deep and superficial *Staphylococcus* infections (Table 6).

**Table 6:** Comparison of infection sites with type of bacterial culture

Bacteria culture	Infection site				Chi-square (p-value)
	Deep		Superficial		
	Number	%	Number	%	
Pseudomonas	1	33.3%	2	28.6%	41.90 (0.01)
Staphylococcus	2	66.7%	5	71.4%	

## Discussion

The WHO has classified obesity in terms of body mass index (BMI). Overweight people have a BMI of between 25 and 29.9 kg/m^2^, obese people have a BMI of between 30 and 40 kg/m^2^, and morbidly obese people have a BMI of over 40 kg/m^2^. Patients who are obese and have spine surgery have been linked to longer operating times, more blood loss, higher treatment costs, a higher chance of dying, and higher rates of venous thromboembolism and surgical site infection [6]. Numerous studies have found a correlation between higher rates of postoperative infection and the presence of obesity, particularly morbid obesity [6,16-18]. In spine surgery, closure methods are usually chosen by the surgeon; however, the literature has little to no consensus regarding particular forms of closure [9].

There is very sparse literature available on the use of different materials for skin closure in orthopedic or spine surgery. According to a thorough analysis by Krishnan et al. [19], individuals who had knee or hip surgery did not significantly vary in terms of secondary outcomes or superficial infection between sutures and staples.

In a meta-analysis encompassing non-orthopedic disorders, Iavazzo et al. [20] discovered that staples led to a reduced rate of wound infections and a quicker wound closure time. However, compared to subcuticular closure using 2-octyl cyanoacrylate glue, Ando et al. [8] discovered that stapled wounds following posterior spine fusion had a noticeably greater prevalence of wound infection. There is weak evidence and a lot of heterogeneity in each of these trials. The most recent information on the best wound closure mechanism needs to be looked at because various surgical procedures might benefit from different wound closure mechanisms [9].

Our study included obese patients who underwent lumbar spine surgeries, as they are more prone to postoperative complications such as surgical site infections and venous thromboembolism [21]. They are also a high-risk population, as they are more prone to disc degeneration and low back pain [22]. However, systematic reviews on the effects of obesity on spinal surgeries noted that there was equivalent or higher postoperative treatment efficacy in such cases and recommended strategies to reduce postoperative complications [21,23]. Wound closure techniques have evolved over the years for better wound management of simple to complex wounds [24]. Sutures, staples, tissue adhesives, adhesive tapes, surgical zippers, and laser-assisted tissue bonding are a few of the materials used worldwide for the closure of wounds. Each of these materials has its advantages and disadvantages, and hence, there is a need to study these materials for different surgical techniques and populations to provide evidence-based optimum wound management [9,22]. In the current study, we examined two such wound management approaches (absorptive sutures and staples) in obese patients following single-level posterior lumbar decompression surgery. As far as we know, no studies have been conducted to compare different skin closure techniques used during lumbar spine surgeries, particularly on obese patients.

There was no discernible variation in the groups' mean age or gender distribution (p>0.05). The groups' blood parameters do not differ significantly from one another. The mean amount of time that patients have developed SSI varies significantly between groups (p<0.05).

There is a significant difference in the presence of SSI between the groups. There is a significantly higher incidence of SSI in the absorbable suture group (35%) compared to the staple group (15%). Also, there was a higher incidence of *Staphylococcus* infection in the suture group compared to the staple group. This was similar to a study done on the general population with lumbar spine surgery by Ando et al. [8], where 2.7% of patients in the staple technique group had SSI and 0% of cases in the suture technique group (2-octyl cyanoacrylate) had SSI. The mean duration of development of SSI in the absorbable suture group (9.86±2.12) was early compared to the staple group (12.67±2.08) (p<0.05).

Culture reports indicated that the presence of *Pseudomonas* was observed in deep infections, and *Staphylococcus* infection was significantly higher in superficial infections. These findings are in line with another study by Smith et al. [14] that discovered that treating wounds with staples as opposed to sutures significantly increases the incidence of wound infection. After hip surgery, the chance of getting a wound infection was four times higher with staple closure than with suture closure [13].

Metal staples have traditionally been considered a more costly wound closure option than suturing wounds; nevertheless, quicker recovery times and easier clip removal may reduce these costs [13].

Nevertheless, the advantages of using absorbable sutures for wound closure include fewer procedure-related discomforts and time savings. These monomers form a hard barrier that sloughs off when the wound heals, saving patients and medical staff time and money by eliminating the need to remove a non-absorbable suture. The advantage of staplers is their quick closure of the wound, with minimal technique required for the closure.

Although there might not be a big difference in the way staples and sutures are used for skin closure, it is still important to think about the logistical and financial outcomes. A few studies have measured and compared the closure time taken using sutures and staples for skin closure in various surgeries [25,26]. The use of staples for skin closure was found to save an estimated seven minutes of time, according to Slade Shantz et al. [25]. Staples could potentially reduce overscheduling and hospital expenditures by an estimated 35 minutes of operating time if an orthopedic surgeon performed five procedures a day on average [25]. Similar results were observed in another study by Liew and Haw [26], who concluded that staples significantly reduce the operative time.

Graham et al. [27] proposed a direct relationship between wound collagen deposition and wound oxygenation and perfusion. They found that, when compared to the suture group, wounds closed with staples had considerably higher blood contact in the wound at seven days and more favorable blood perfusion metrics (0.02).

Murphy et al. [28] blamed a subpar staple placement technique for the subpar staple results. The correctness of the dermal margin coaptation can be affected by the type of closure and the accuracy of the suture or staple closure. Less-than-optimal healing outcomes can result from inadequate practice.

Our pragmatic study showed better results in terms of wound healing and infection rates with the use of staples compared to sutures in obese patients who are already at risk for postoperative wound infections.

Limitations

Our study is not devoid of limitations. One of the major limitations of the study is its record-based, retrospective nature. Hence, future prospective randomized controlled trials may provide additional evidence-based knowledge on the subject. However, due to the time-bound nature of the study and good documentation practices in the study area, this limitation was reduced. The other limitation was small sample size, which could limit our ability to draw definitive conclusions. Also, comorbidities such as diabetes mellitus, which could have influenced the rate of infection, were not considered.

## Conclusions

Compared to wound closure with absorbable sutures, the current study showed a decreased incidence of surgical site infection in individuals receiving skin staples. Also, the duration of infection development was longer in the staple group compared to the suture group. With the advantage of quick closure and a lower infection rate, wound closure in obese individuals with a skin stapler is safer and more promising.
